# A Rare Case of Acute Pericarditis Due to SARS-CoV-2 Managed With Aspirin and Colchicine

**DOI:** 10.7759/cureus.12534

**Published:** 2021-01-06

**Authors:** Komandur Thrupthi, Adithya Ganti, Trishna Acherjee, Maham A Mehmood, Trupti Vakde

**Affiliations:** 1 Internal Medicine, BronxCare Health System, Bronx, USA; 2 Pulmonary and Critical Care, BronxCare Health System, Bronx, USA

**Keywords:** viral pericarditis, covid 19, covid-19 pneumonia

## Abstract

SARS-CoV-2 infection presents with predominant respiratory illness. Cardiac injury has been reported in patients with SARS-CoV-2 infection. The spectrum of cardiac involvement ranges from pericarditis to myocarditis. Acute pericarditis attributed to SARS-CoV-2 is rare.

A 68-year-old male with co-morbid condition of hypertension and arthritis presented with chest tightness, cough and exertional shortness of breath for five days. He was tachycardic at the time of presentation and cardiac auscultation was positive for pericardial rub. His room air oxygen saturation was 95%. Chest imaging studies revealed bilateral infiltrate. His electrocardiogram showed ST elevation with diffusely elevated J point in lead II, III, aVF and V4-V6. Echocardiogram was unrevealing for pericardial effusion and left ventricular ejection fraction was normal. Serial troponin level did not reveal a rising trend. The nasopharyngeal swab was positive for SARS-CoV-2 RNA. Nonsteroidal anti-inflammatory drugs (NSAIDs) use in SARS-CoV-2 positive patient is debatable. The patient had acute pericarditis due to SARS-CoV-2 and it was treated with high dose aspirin with colchicine.

Acute pericarditis is a rare complication of SARS-CoV-2 infection and can be managed with aspirin and colchicine.

## Introduction

Hubei province in China first reported the atypical pneumonia of unknown origin. The etiology of pneumonia revealed to be a novel virus and was named coronavirus 2019 (2019-nCoV), also known as COVID-19, by the health commission of China. It is genetically similar to severe acute respiratory syndrome (SARS) Coronavirus of 2002 and hence the WHO termed it SARS-CoV-2 [1].

SARS-CoV-2 disease is a global pandemic with a predominant pulmonary manifestation. SARS-CoV-2 generally presents with viral prodromal symptoms and patients with pre-existing co-morbid conditions like hypertension, diabetes mellitus, coronary artery disease have an adverse outcome. There is ample evidence to opine on gastrointestinal, hepatic, cardiac, and central nervous system involvement of SARS-CoV-2. It is estimated that around 20%, of infected, may have a cardiac injury related to SARS-CoV-2 [2]. The spectrum of cardiac involvement ranges from myocarditis with left ventricular dysfunction to pericarditis [3]. It is challenging to opine on the exact incidence of pericarditis based on a review of current literature. The majority of evidence of SARS-CoV-2-related pericarditis is based on case reports and it is one of the rare findings of SARS-CoV-2 [4]. Here we present a case of SARS-CoV-2 pneumonia who also reported chest pain and was diagnosed with acute pericarditis. Nonsteroidal anti-inflammatory drugs (NSAID) are first-line therapy for the management of acute pericarditis. The emergence of evidence, though uncertain, of NSAID-related worsening of SARS-CoV-2 pneumonia led us to manage pericarditis with colchicine. Our case report demonstrates a rare presentation of SARS-CoV-2 related to acute pericarditis and the utility of colchicine in its management.

## Case presentation

A 68-year-old male with a past medical history of hypertension and osteoarthritis presented to the emergency department with a chief complaint of chest tightness for five previous days. The patient also reported having a dry cough, mild fatigue, and shortness of breath on exertion. On further evaluation, the patient revealed that his chest pain is sharp, stabbing type with no radiation, persistent with no respiratory variation, and exacerbation of pain on bending forward. He denied any pain on exertion, any prior episodes of chest pain, COVID-19 positive sick contact, and prior history of any rheumatological disorder.

**Figure 1:**
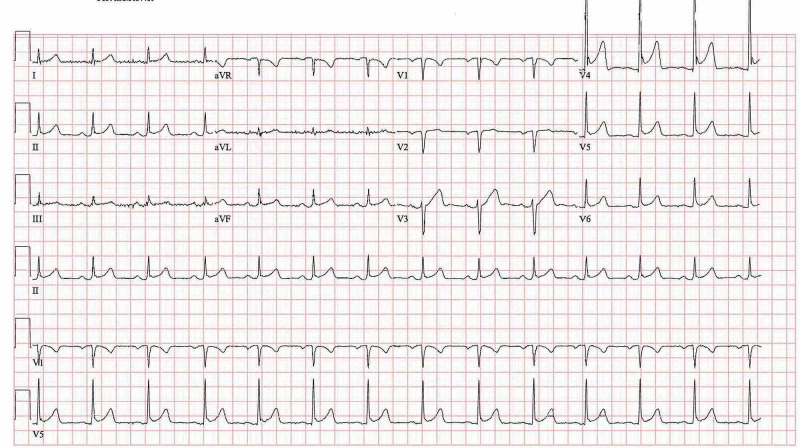
Electrocardiogram demonstrates ST-elevation in I, II, III, aVF and V4-V6 with J point elevation suggestive of acute pericarditis

**Figure 2:**
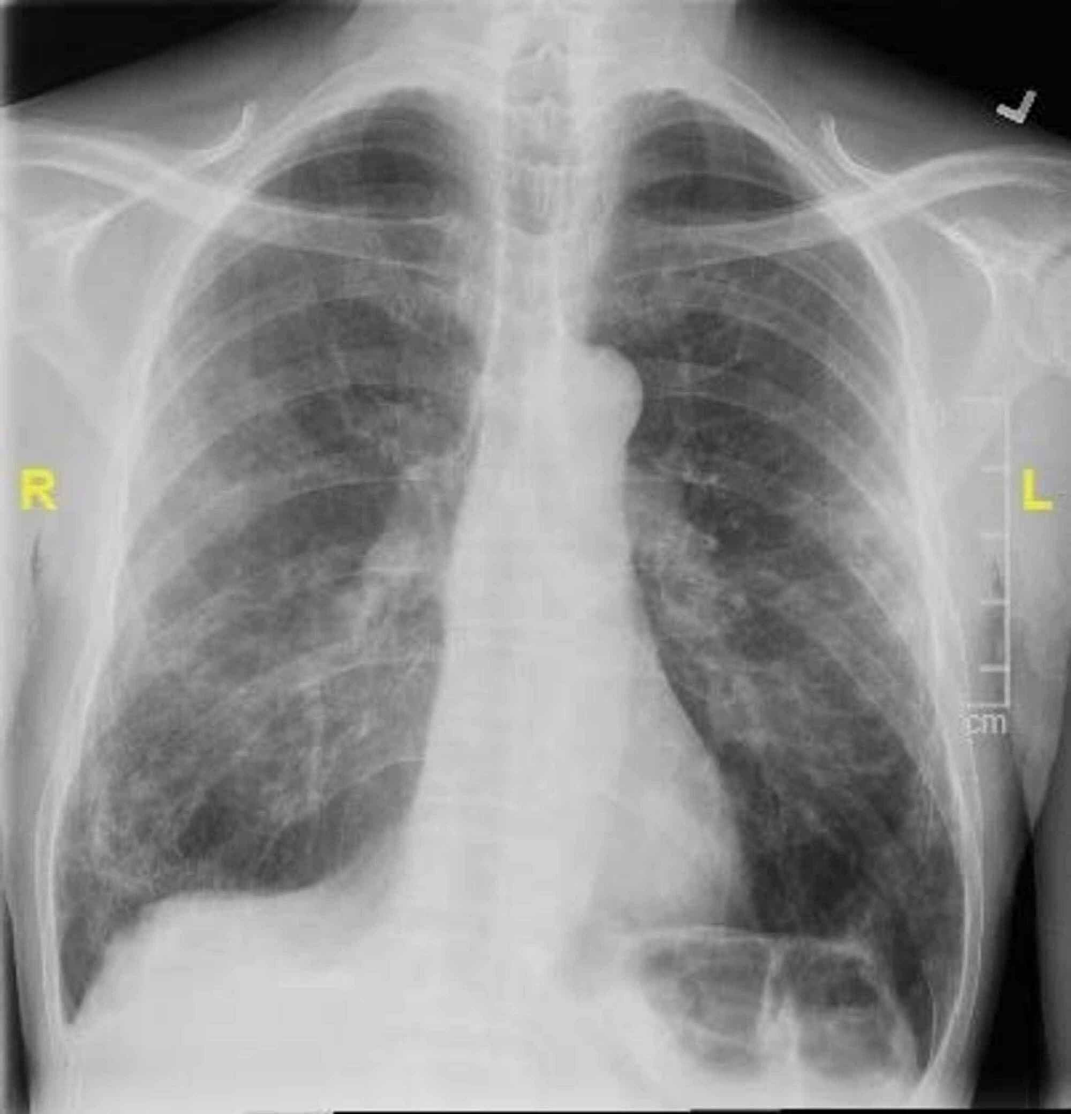
X-ray chest demonstrates bilateral opacities

**Figure 3:**
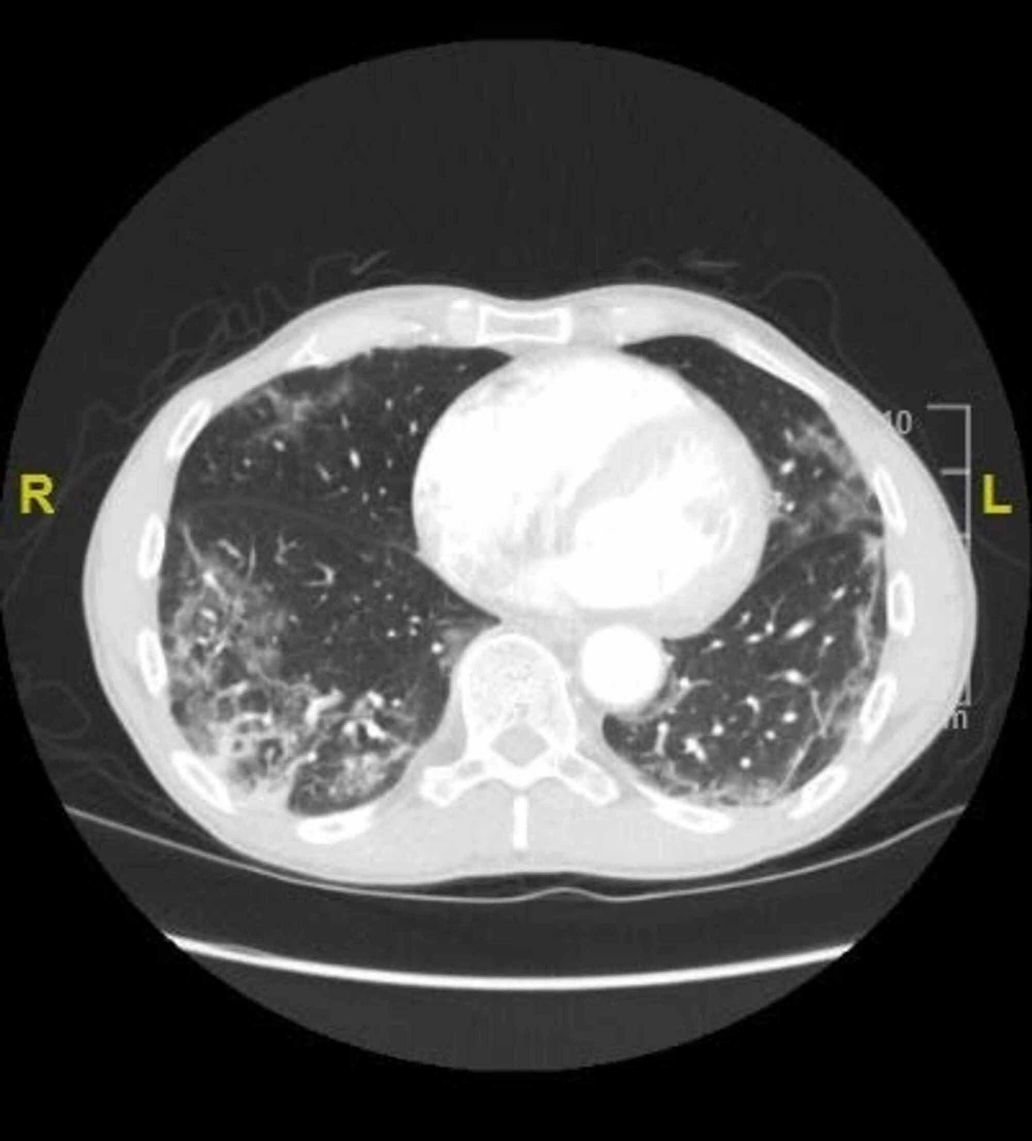
Cross-section of the CT-chest revealing extensive peripheral patchy ground glass opacities

On examination, the patient was afebrile with a mean arterial pressure of 93 mm Hg, heart rate of 125 beats per minute, oxygen saturation of 95% on room air. The physical exam was significant for a pericardial rub and bilateral extensive expiratory crackles. His troponin on the day of presentation was 17 ng/L with the NT-proB-type natriuretic peptides (BNP) of 54 pg/ml. Another pertinent laboratory parameter is demonstrated in Table 1. A 12-lead electrocardiogram revealed an ST elevation in leads II, III, AVF, and V4-V6, with diffusely elevated J point (Figure 1). Findings on the initial X-ray of the chest revealed bilateral diffuse patchy and peripheral interstitial infiltrates (Figure 2). A computerized tomographic pulmonary angiogram (CTPA) did not reveal any pulmonary arterial filling defect. However, it revealed extensive peripheral airspace disease (Figure 3).

**Table 1:** Laboratory parameter at presentation and interval follow-up WBC: White blood cell; ESR: Erythrocyte sedimentation rate; LDH: Lactate dehydrogenase; CRP: C-reactive protein.

LABORATORY FINDINGS	Day-1	Day-2
WBC	4.5	5.9
Lymphocyte count	0.9	0.9
ESR	89	
D-dimer	486	
LDH	338	
CRP	38.7	21.90
Ferritin	1733	993
Troponin	16.17	12

The patient was considered to be a COVID-19 suspect (patient under investigation) with pneumonia and possible myopericarditis. Transthoracic echocardiography revealed a 69% ejection fraction with no pericardial effusion. There were no wall motion abnormalities on the echocardiogram. The nasopharyngeal swab was performed for Pan-SARS and SARS-CoV-2 RNA. The enzyme-linked immunosorbent assay (ELISA) was reported positive for SARS-CoV-2 RNA and negative for Pan-SARS panel. Other pertinent laboratory parameters including urine Legionella and Mycoplasma pneumonia IgM were unrevealing for positive findings.

The patient’s serial troponins were followed every 8 hours and noted to be normalized in 24 hours of presentation to 12 ng/L. Given the non-rising trend of troponin level, absence of myocardial wall motion abnormality, typical pericardial chest pain, and presence of pericardial rub, the patient was managed as myocarditis. The recommendations prevalent in our institution were followed for the management of COVID-19 pneumonia. He was initiated on doxycycline 100 mg orally twice daily and amoxicillin-clavulanate 875 mg-125 mg twice daily orally. The patient’s hospital course was stable and he remained afebrile. He was initiated on aspirin 650 mg tablet three-time daily with colchicine 0.6 mg twice daily. The patient was not in respiratory distress, was saturating 95% on room air. SARS-CoV-2 was presumed to be a possible etiology for his acute pericarditis and was planned for rheumatology evaluation on an ambulatory basis. The patient was not given steroids during hospitalization as he responded well with aspirin and colchicine. The patient was followed with a telehealth visit and reported resolution of symptoms three weeks after discharge. He was planned for the outpatient cardiology appointment but failed to follow up.

## Discussion

The SARS-CoV-2 virus transmits through respiratory droplets, with an incubation period of five days to 10 days [5]. SARS-CoV-2 binds to angiotensin-converting enzyme type-2 (ACE-2) receptors of host cells and after internalization, it leads to the downregulation of ACE2 receptors [6]. ACE2 is expressed in the lungs, kidneys, heart, and blood vessels and is the key enzyme to regulate angiotensin II and angiotensin I. There is a better understanding of the life cycle of SAR-CoV-2 and most of the clinical manifestation can be from the viral replication or host response [7].

The majority of the patients with the SARS-CoV-2 have a mild to moderate clinical presentation [8]. The severe presentations have predominant pulmonary involvement. In our case report, CT imaging was not used as a tool to assess the severity of the disease but it was mainly based on clinical presentation and response to treatment. Cardiac complication from SARS-CoV-2 has given insight into another major predictor of mortality. In a cohort of 466 patients in Wuhan, the presence of cardiac injury, as diagnosed by elevated serum troponin levels, had 51.2% mortality versus 4.5% in those who did not have cardiac injury [9]. Acute myopericarditis with subsequent arrhythmia or decrease in left ventricular function may be the pathophysiology for SARS-CoV-2-related cardiac morbidity [10].

Acute pericarditis is diagnosed if the patient has two of four presenting clinical criteria [11]. These criteria are: i) chest pain, ii) auscultation findings of pericardial friction-rub, iii) ECG findings suggestive of pericarditis, and iv) pericardial effusion. Our patient had all characteristics except pericardial effusion. However, pericardial effusion is present only in up to 60% of patients [12]. Serial troponins and myocardial dysfunction will aid in assessing myocarditis in conjunction with pericarditis [13]. There was no wall motion abnormality or low ejection fraction on the echocardiogram in our patient. Hence, the cardiac manifestation in our patient was likely due to acute pericarditis.

A viral infection is the most common etiology for acute pericarditis. Pericardial inflammation due to viral replication or immune response explains the pathogenesis of acute pericarditis [14]. Nonsteroidal anti-inflammatory drugs (NSAIDs) are the first line in pharmacological treatment for acute pericarditis. The association of NSAIDs and the worsening of COVID-19 is not clear but should be avoided if possible [15]. Colchicine used for recurrent pericarditis can be utilized in acute presentation as well [13, 16]. We performed an ‘All Field’ PubMed search with "covid 19” AND "pericarditis" and reviewed nine articles as of May 21, 2020. We did not find the use of Colchicine in the treatment of SARS-CoV-2 pericarditis. The anti-inflammatory potency of Colchicine is being evaluated for COVID-19 at this time [17]. However, our case report demonstrates the utility of managing SARS-CoV-2 pericarditis with Colchicine.

## Conclusions

Patients with SARS-CoV-2 infection can have a rare presentation with acute pericarditis. Echocardiogram should be performed to assess wall motion abnormality along with left ventricular ejection fraction to rule out myocardial involvement. SARS-CoV-2 pericarditis can be treated with Colchicine.
